# Behavioural and physiological responses to prey-related cues reflect higher competitiveness of invasive vs. native ladybirds

**DOI:** 10.1038/s41598-017-03471-9

**Published:** 2017-06-16

**Authors:** Gabriele Rondoni, Fulvio Ielo, Carlo Ricci, Eric Conti

**Affiliations:** Department of Agricultural, Food and Environmental Sciences, University of Perugia, Borgo XX Giugno, 74, 06121 Perugia, Italy

## Abstract

Understanding the traits that might be linked with biological invasions represents a great challenge for preventing non-target effects on local biodiversity. In predatory insects, the ability to exploit habitats for oviposition and the physiological response to prey availability differs between species. Those species that respond more readily to environmental changes may confer to their offspring a competitive advantage over other species. Here, we tested the hypothesis that the invasive *Harmonia axyridis* (Coleoptera: Coccinellidae) makes better use of information from a plant-prey (*Vicia faba* - *Aphis fabae*) system compared to the native *Oenopia conglobata*. Y-tube olfactometer bioassays revealed that both species used olfactory cues from the system, but *H. axyridis* exhibited a more complete response. This species was also attracted by plants previously infested by aphids, indicating the capacity to exploit volatile synomones induced in plants by aphid attack. Oocyte resorption was investigated when different olfactory stimuli were provided under prey shortage and the readiness of new oogenesis was measured when prey was available again*. H. axyridis* exhibited higher plasticity in oogenesis related to the presence/absence of plant-aphid volatiles. Our results support the hypothesis that *H. axyridis* is more reactive than *O. conglobata* to olfactory cues from the plant-prey system.

## Introduction

With increased globalization, several exotic species are introduced into new areas every year, either accidentally or intentionally. Some species do not find suitable conditions, while others successfully establish and, as in the case of natural enemies that are introduced within accurate biological control programs, show beneficial effects on local biodiversity, economic activities and the environment. However, many non-native species, because they are injurious to crops or human and animal health in their native area, or because they are natural enemies that were improperly or accidentally introduced, may become especially invasive (invasive alien species - IAS) and may greatly threaten biodiversity, health and economy^1^. It has been estimated that from 1906 to 1991, 79 exotic species caused $97 billion damage to the economy of the United States^2^.

Some common traits, intrinsic to the species, are consistently related to the successful establishment of non-native organisms. Among plants, a recent meta-analysis underlined that invasive species have traits associated with higher performance over non-invasive species^3^. The biomass is generally greater and the trade-off between growth and reproduction favours higher reproductive input in invasive plants compared to native plants^3^. For freshwater fishes, the tolerance to extreme temperatures and to changes in water quality, a large body size and a wide distribution in the native range have all been linked to establishment success and are considered robust indicators of it^4, 5^. Similarly, invasion success by arthropods can be linked to reproductive strategies and resource acquisition. In their review paper, Comont *et al*.^6^ showed that ladybird species with broad diet range are likely to have a wider distribution than species with a more limited prey range^6^. For all of the above-mentioned taxa, in particular invertebrates, attributes of the invaded community, such as low community maturity and high niche opportunities, may play an important role in fostering invasion^7^. Additionally, the lack of regulation by natural enemies in the invaded area (i.e., the enemy release hypothesis; ERH)^8^ and the consequent allocation of energy resources to fitness rather than defences (i.e., the evolution of increased competitive ability; EICA)^9^ have been proposed to be the main determinants in the invasion success. Among insects, ladybird beetles may represent valuable model species to study different patterns of invasive processes^10^. Invading ladybirds invest high resources in reproduction and tend to show a large body size and high genetic diversity, phenotypic plasticity and likelihood to engage in competitive interactions with non-invading species^11, 12^. Here, we focus on some aspects of the oviposition behaviour and reproductive physiology of ladybird beetles. The optimal oviposition theory predicts that females of predatory ladybird beetles would lay their eggs accordingly to habitat quality, prey abundance and suitability and the risk of competition with other predators, all of which affect offspring survival and development^13–15^. Although several studies have focused on comparing characteristics that make a patch “suitable” for a certain species, very few have focused on the ability of a female to acquire the information from the habitat and use it to locate a suitable oviposition site^16^. Theory predicts that ladybird species might exhibit differences in behaviour and ability to exploit a habitat for oviposition^17^. Species that respond more readily to an environment of good quality might confer to their offspring an advantage over juveniles of other competitors^18^. In fact, developing larvae might exploit preys that are free from competitors, allowing time to increase their body weight until the arrival of other predators^19^. This results in a competitive advantage, especially in the case of intraguild predation events due to an occasional shortage of the essential prey^20^.

The exotic predator *Harmonia axyridis* (Pallas) (Coleoptera: Coccinellidae) is native to Asia and was widely introduced as a biological control agent of pest aphids, but it has spread also to many Countries where it was never intentionally released (reviewed by ref. 21). Due to intrinsic traits such as large body size, high voracity and high reproductive investment, *H. axyridis* has often outcompeted populations of native predatory ladybird beetles to become the most represented species^22–24^. *H. axyridis* can dominate the coccinellid community in invaded areas, affecting the native community through juvenile competition for resources, intraguild predation, and escape from natural enemies^10, 21, 25–27^. These factors also have been shown to promote invasiveness and might be a consequence of (1) an intrinsic higher ability of *H. axyridis* against competitors^28^ and (2) a maternally driven decision in habitat choice for oviposition, which might indirectly confer a competitive advantage to offspring.

Despite the relevant literature behind *H. axyridis*, very little is known about its ability to exploit habitats for oviposition. We addressed this aspect here for this invasive species and for native *Oenopia conglobata* (L.) (Coleoptera: Coccinellidae). *O. conglobata* is potentially displaced by *H. axyridis* ^29^ because of its low competitive ability in direct intraguild contests^30, 31^ or field conditions^32^. Both ladybird beetles should be considered generalists, since their diet range largely extends to species other than aphids^13^. Both ladybirds also feed on psyllids and coccids, and *O. conglobata* is also frequently observed to prey on juvenile stages of leaf beetles, such as *Plagiodera versicolor* Laich. on willows. Additionally, both ladybird species have a preference for trees but may also occur on herbaceous plants^33, 34^. In our experiments, we chose the system represented by *Aphis fabae* Scop. developing on *Vicia faba* L., as *A. fabae* represents, for both predators, a very suitable prey compared with other common aphids in terms of survival, larval growth, development and adult fresh weight^35, 36^. We tested the prediction that *H. axyridis*, as more competitive than *O. conglobata*, makes better use of information from prey and from plants attacked by prey for a first assessment of patch quality. We performed a first group of experiments in a Y-tube olfactometer to test whether selected olfactory stimuli trigger the behavioural attraction of coccinellid females. The specific aim was to test whether odours emitted by the aphid, the plant and their interaction might represent kairomones or synomones, respectively, for the predators. Natural enemies are known to rely on a series of chemical cues used for long-range orientation to efficiently invest their limited time in the location of hosts and preys (reviewed by ref. 37).

In a second group of experiments, we tested whether odour stimuli from prey-infested plants induce a physiological response in the reproductive investment of the predators, thus influencing oocyte resorption. Oosorption occurs with the occurrence of an unfavourable environment, including food shortage and starvation^38–40^. Under these conditions, ladybird females resorb nutrients from developing oocytes, investing resources in survival rather than reproduction. However, it is not clear how they respond to prey-related volatiles. A link between poor environmental conditions and oosorption has been previously observed^40^. Therefore, we hypothesized that ladybirds might use volatile cues to assess the perceived quality of the habitat and modify ovarian development accordingly.

## Results

### Behavioural observations

**Figure 1:**
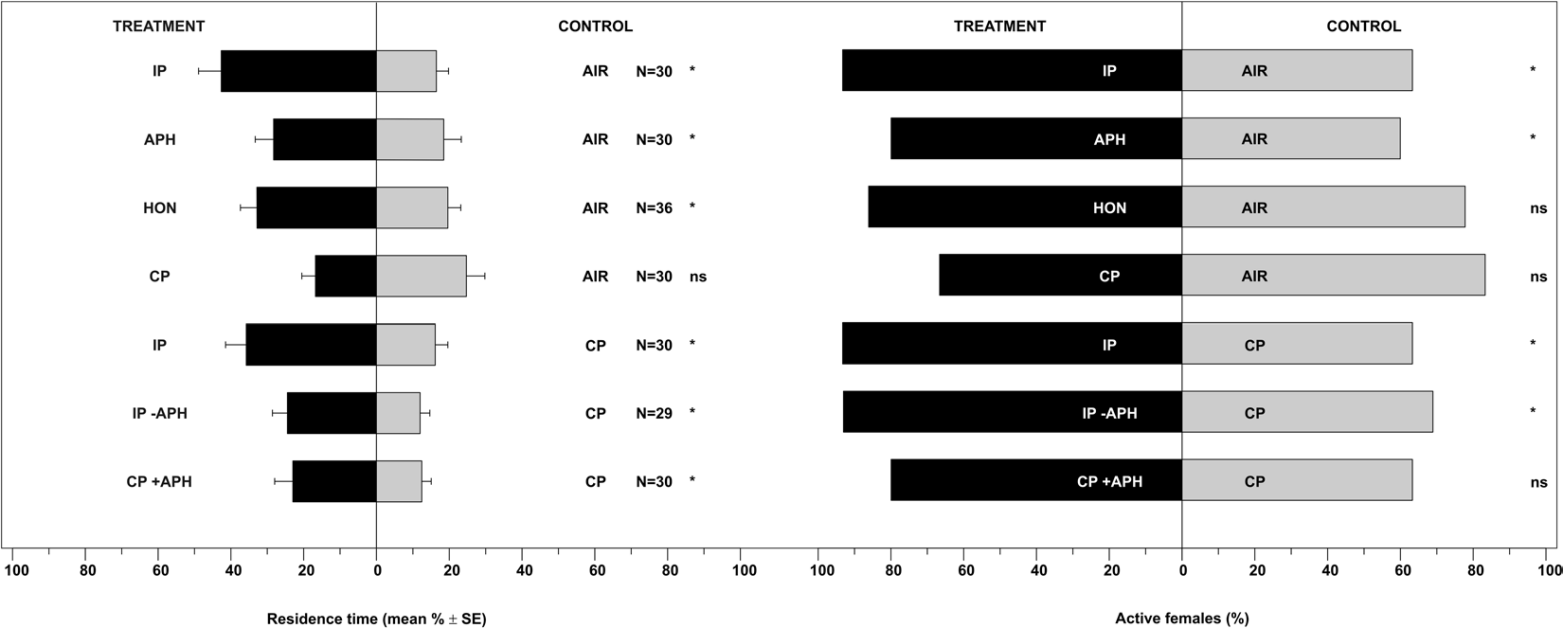
Residence time and active *Harmonia axyridis* females in each arm of the olfactometer connected with volatiles from the *Vicia faba* – *Aphis fabae* system. Odour treatments consisted of a *V. faba* plant infested with *A. fabae* colonies (IP); juvenile and adult *A. fabae* individuals (APH); honeydew from *A. fabae* individuals (HON); a clean *V. faba* plant (CP); a *V. faba* plant infested with *A. fabae* colonies but with aphids carefully removed prior to the bioassay (IP -APH); and a clean *V. faba* plant plus *A. fabae* individuals separated from the plant (CP + APH). Control consisted of either a stream of air (AIR) or a clean *V. faba* plant (CP). N = number of replicates. * = P < 0.05; ns = not significant.

**Figure 2:**
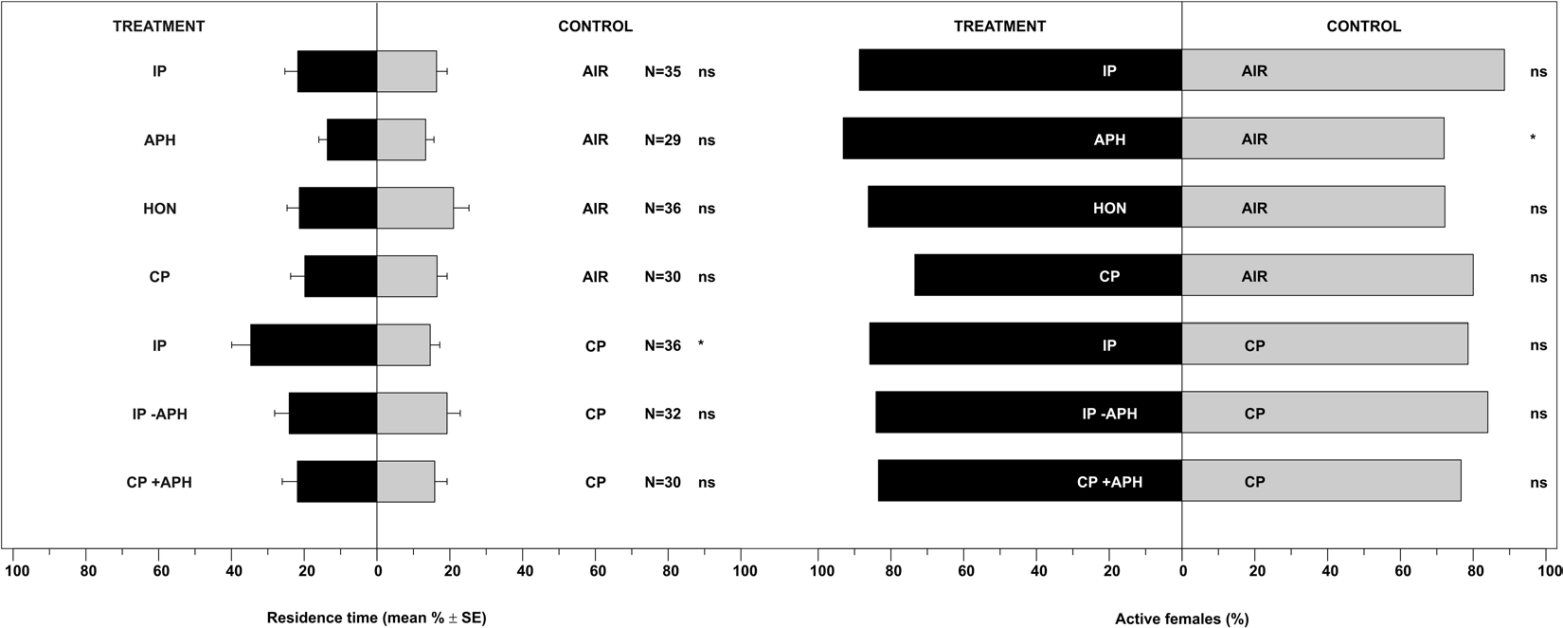
Residence time and active *Oenopia conglobata* females in each arm of the olfactometer connected with volatiles from the *Vicia faba* – *Aphis fabae* system. Abbreviations as in Fig. 1. N = number of replicates. * = P < 0.05; ns = not significant.

*Harmonia axyridis* females were significantly attracted to volatiles emitted by plants infested by *A. fabae*, or to aphids alone or to honeydew (Fig. 1, Table S1: P < 0.05 for all the comparisons) compared to clean air. Neither treatment condition was attractive for *O. conglobata* (Fig. 2, Table S1: P > 0.05 for all the comparisons). A clean plant did not stimulate a significant response in *H. axyridis* (Fig. 1, Table S1: P = 0.956) or *O. conglobata* (Fig. 2, Table S1: P = 0.296). Compared to a clean plant, *H. axyridis* and *O. conglobata* were significantly attracted to a plant infested with *A. fabae* (*H. axyridis*: Fig. 1, Table S3: P < 0.001; *O. conglobata*: Fig. 2, Table S3: P = 0.002). *H. axyridis* was also attracted to a plant from which aphids were removed prior to the bioassay or from a clean plant with aphids kept separate (Fig. 1, Table S3: P < 0.05 for all the comparisons), while *O. conglobata* was not (Fig. 2, Table S3: P > 0.05 for all the comparisons). Data on active females confirmed the response of *H. axyridis* to the infested plant vs. air (Fig. 1, Table S2: P = 0.006) or vs. clean plant (Fig. 1, Table S4: P = 0.006) and to a plant from which aphids were removed prior to the bioassay (Fig. 1, Table S4: P = 0.015). More *O. conglobata* females moved to aphids (Fig. 2, Table S2, P = 0.026), whereas all other treatments were not significantly different from controls (P > 0.05).

**Figure 3:**
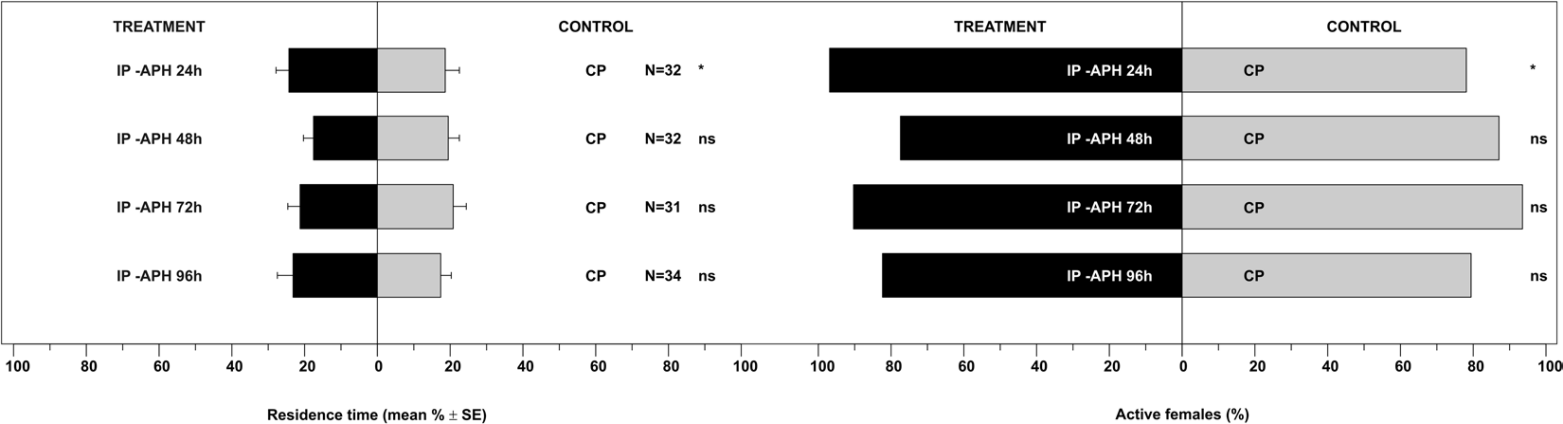
Residence time and active *Harmonia axyridis* females in each arm of the olfactometer connected with volatiles from the *Vicia faba* – *Aphis fabae* system. Odour treatments consisted of a *V. faba* plant infested with *A. fabae* colonies but with aphids carefully removed at 24 (IP -APH 24 h), 48 (IP -APH 48 h), 72 (IP -APH 72 h) or 96 (IP -APH 96 h) hours prior to the bioassay. Control consisted of a clean *V. faba* plant (CP). N = number of replicates. * = P < 0.05; ns = not significant.

When plants were exposed to *H. axyridis* at different time intervals after aphid removal, they maintained their attractiveness 24 h after the end of the infestation (Fig. 3, Table S5: P = 0.03). Whereas plants were not significantly attractive after 48, 72 or 96 h (Fig. 3, Table S5: P > 0.05 for all comparisons), compared to the controls. Data on active females confirmed *H. axyridis* response (Fig. 3, Table S6: P = 0.025 at 24 h; P > 0.05 at 48, 72 or 96 h).

**Figure 4:**
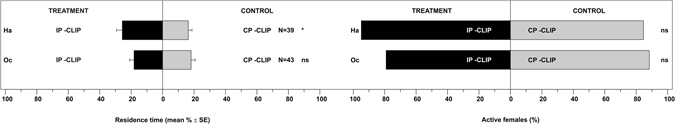
Residence time and active *Harmonia axyridis* and *Oenopia conglobata* females in each arm of the olfactometer connected with volatiles from the *Vicia faba* – *Aphis fabae* system. Odour treatments consisted of a locally infested plant obtained by exposing the surface of the 4th leaf to juvenile and adult aphids inside a clip cage (IP -CLIP). Control consisted of a clean *V. faba* plant with an untreated 4th leaf with a clip cage (CP -CLIP). Caged leaves were removed prior to the bioassays. N = number of replicates. * = P < 0.05; ns = not significant.

Plants that were infested locally (i.e., on a single leaf), after the removal of the infested leaf maintained their attractiveness toward *H. axyridis* (Fig. 4, Table S7: P = 0.008) but not toward *O. conglobata* (Fig. 4, Table S7: P = 0.538). No differences were detected in the numbers of *H. axyridis* or *O. conglobata* females that moved to the two olfactometer arms (Fig. 4, Table S8: P > 0.05 for all comparisons).

### Ovarian dynamics

The best-fit model to explain ovarian development retained the principal effect of the coccinellid species, the plant treatment, the time post-set-up of the experiment, and the interaction between time and species (Table 1). The model exhibited the lowest AIC (44.32) and was not significantly worse than the full model (AIC = 50.14). Immature oocytes were significantly less numerous when ladybirds were exposed to odours from an aphid-infested plant (Table 1: model coefficient −0.10 ± 0.05, z = 2.24, P = 0.028; Supplementary Figure S1) and were less numerous in *O. conglobata* than in *H. axyridis* (Table 1: model coefficient −0.19 ± 0.07, z = −2.77, P = 0.007). Increasing the time since set-up of the experiment led to a significant negative effect on the maturity of oocytes (Table 1: model coefficient −0.024 ± 0.002, z = −11.91, P < 0.001). When the ladybirds were fed with aphids after a starvation period, feeding led to an increasing maturation of oocytes (Supplementary Table S9: model coefficient at 24 h 0.46 ± 0.10, t = 4.49, P < 0.001). After 24 h of feeding, mature oocytes were lower in dissected *O. conglobata* females compared to *H. axyridis* (model coefficient −0.30 ± 0.14, t = −2.13, P = 0.038), whereas no significant differences were observed after 48 h (model coefficient −0.09 ± 0.14, t = ,−0.66, P = 0.515).

**Table 1:** Regression coefficients, standard errors and significance for variables retained in the best-fit model describing the relationship between ovarian resorption and (i) plant treatment (clean vs. infested); (ii) time from set-up (0, 6, 12, 24, 48 h); (iii) coccinellid species (*Harmonia axyridis* or *Oenopia conglobata*); and (iv) interaction between time and coccinellid species.

Variable	(Level)	Coeff	SE (coeff)	P
Intercept		0.53	0.05	<0.001
Plant	(infested plant)	−0.10	0.05	0.028
Time		0.024	0.002	<0.001
Species	(*O. conglobata*)	−0.19	0.07	0.007
Time: species		0.01	0.00	0.038

## Discussion

Volatile cues associated with the plant-aphid system have been shown to elicit a process of long-range host location for parasitoids^41^ as well as predators^42^. For insect predators such as ladybird beetles, the choice of an optimal site to lay eggs is a crucial step that is important for the survival of their offspring^17^. In our study, *H. axyridis* showed a higher response than *O. conglobata* to olfactory cues associated with a plant-aphid system, both at the behavioural and physiological level.

The strong response of *H. axyridis* to aphid-infested plants suggests that at the aphid density chosen in our bioassay, this species is able to obtain information from volatile compounds. We observed that aphid and honeydew odours might act as a kairomonal message used by *H. axyridis* to locate prey. For this species, our results are consistent with those obtained by Leroy^43^, who identified a positive response to volatiles emitted from the aphid *Megoura viciae* Buckton, including Z, E-nepetalactone, [E]-β-farnesene, α-pinene and β-pinene, and 3-hydroxy-2-butanone, 3-methyl-butanal, 3-methyl-1-butanol and limonene emitted from aphid honeydew. In describing the mechanism of prey finding in *H. axyridis*, Obata^44, 45^ demonstrated the involvement of visual and olfactory cues, in particular the attractiveness of odours from the infested plants and the odour from aphids combined with visual cues from a clean leaf. In our experiment, when the aphids and honeydew were removed from a *V. faba* plant, the plant maintained its attractiveness, suggesting the involvement of synomones emitted as a consequence of aphid feeding. Such synomones are systemically induced, as indicated by the attractiveness of *H. axyridis* to *V. faba* plants that were infested locally after removal of the infested leaf. The existence of induced volatiles emitted from plants that may be responsible for the attraction of *H. axyridis* females has only been documented with (Z)-3-hexenol. This compound was produced in nettle plants that were mechanically damaged to tentatively simulate aphid infestation^46^, but probably (Z)-3-hexenol is not a reliable indicator of aphid infestation. In fact, an increase in its emission has been linked to damage from chewing herbivores also (reviewed by ref. 47), suggesting that *H. axyridis* responds to non-specific cues because its broad diet range. A further explanation for *H. axyridis* attraction is that *V. faba* plants infested by *A. fabae* produce one or more specific compounds (herbivore-induced synomone) that might be reliable indicators of the aphid presence, but such compounds have to be identified.

By contrast, *O. conglobata* showed significant attractiveness only to the treatment represented by aphids or by aphid-infested plants when tested vs. clean plants. Generally, this species showed a lower response to odours from aphids and/or plants compared to *H. axyridis*. A possible explanation might be that *O. conglobata* requires a complete set of odour cues from infested plants to assess a suitable habitat for oviposition. Aphids alone are not attractive for *O. conglobata*. Additionally, it appears that this species does not respond to induced synomones, thus explaining the lower ability in patch exploitation compared to *H. axyridis*. Olfactory sensilla used to perceive volatiles in the environment are probably located on the antenna. In *H. axyridis* females, those sensilla possibly include placodea, basiconica and coeloconica^48^. Antennal morphology combined with electrophysiology could reveal differences between *O. conglobata* and *H. axyridis* and possibly explain their different behaviour.

*Harmonia axyridis* is considered responsible for the displacement of native coccinellids in the United States^49^ and Europe^24, 50^. Based on a recent risk assessment conducted on thirty native European ladybird species, the four species *Adalia bipunctata* L., *Adalia decempunctata* (L.), *Calvia decemguttata* (L.) and *O. conglobata* were identified as most at risk following the invasion of *H. axyridis* ^51^. In Belgium, *O. conglobata* exhibits a negative trend in abundance in deciduous trees following the arrival of the invasive *H. axyridis* ^24^. Predation on guild members by *H. axyridis* has been implicated as the primary cause for displacement of native species^10, 28^, and the strength of these interactions has been recently described under open-field conditions^32, 52–54^. Competition for resources has also been hypothesized^10^, and our results provide a partial explanation of this high ability for *H. axyridis*.

In tests of physiological responses, *H. axyridis* exhibited higher plasticity in ovarian dynamics compared to *O. conglobata*. Intriguingly, here we also demonstrated that prey-related volatiles, which provide reliable cues for the presence of prey in the vicinity, are sufficient to induce physiological modifications in ladybirds. Oosorption in *H. axyridis* in the presence and absence of prey was devoted to allocation of nutrients for survival rather than for development, as has been previously described^38^. Here, we found that starved females of both *H. axyridis* and *O. conglobata* are able to modulate egg resorption based on the presence or absence of volatiles from aphids. The presence of prey odours reduced oosorption in both species, but when odours were absent, *H. axyridis* accelerated the resorption process compared to *O. conglobata*. Moreover, *H. axyridis* rapidly produced new eggs once food was provided again. Faster ovarian dynamics, which have also been observed in generalist *vs*. specialist ladybirds^40^, are useful because they enable species to quickly mature oocytes once they locate prey^55^.

In North America, introduced populations of *C. septempunctata* responded more rapidly than the native *Coccinella transversoguttata richardsoni* Brown to changes in prey availability, by engaging in oosorption as a means of reserving resources under poor prey conditions^39^. This behaviour might have promoted the rapid and successful establishment of *C. septempunctata* in North America^39^. Similarly, the greater ability of *H. axyridis* to adjust the rate of oosorption compared to *O. conglobata* could contribute to the abundance and reproductive success of *H. axyridis* in natural and agricultural systems, which exhibit high variation in aphid densities. In this respect our results are in accordance with the greater flexibility hypothesis developed by Baker^56^.

In conclusion, our results support our prediction that *H. axyridis* is more reactive than *O. conglobata* to olfactory cues from the plant-prey system, which might partially explain its higher competitiveness and consequent ability to dominate predator guilds in invaded habitats. Plants may indirectly affect the natural enemy community exploiting their herbivorous pests by mediating multitrophic interactions^57^. In this respect, the possibility of using plant volatiles to manipulate predator behaviour is promising when the purpose is to enhance the biological control of crop pests^58^ or control populations of invasive predators^46^. Therefore, the identification of prey-related volatiles responsible for the attraction of natural enemies would be necessary for a better understanding of prey location mechanisms by invasive vs. native species and for possible field applications.

## Methods

### Plants

Seeds of broad bean plants (*V. faba* cv. Superaguadulce) were immersed for 24 h in a slurry of water and soil (1:4) to favour germination and nodulation with *Rhizobacteria*. The seeds were individually planted in plastic pots (9 × 9 × 13 cm) filled with a mixture of agriperlite, vermiculite, and sand (1:1:1) and grown in an environmental cabinet (24 ± 2 °C, 45 ± 10% RH, 12 h:12 h L:D). Plants were watered daily and, from 1 week post germination, irrigated with an aqueous solution (1.4 g/l) of fertilizer (5-15-45, N-P-K). For the experiments, three week-old broad bean plants with approximately six fully expanded leaves were used.

### Insects

Coccinellid cultures were established from *H. axyridis* and *O. conglobata* adults collected from fields around Perugia (Italy) and reared under controlled conditions (24 ± 2 °C; 70 ± 5% RH; 14 h: 10 h L:D). Bugs were fed on an *ad libitum* diet of broad bean twigs infested with *A. fabae*. Food was changed every 2–3 days, and separate plastic cylindrical cages (Ø = 25 cm, height = 30 cm) covered by a fine tissue mesh to allow ventilation were used for larvae and adults. After the emergence from pupae, male and female ladybirds were isolated for a few days with aphids and then paired in Petri dishes to allow mating. For all bioassays, groups of six physogastric females were detected and individually isolated in small vials 30 min before bioassays and transferred to the bioassay room to be acclimatized. After behavioural observations, females were returned to Petri dishes with *A. fabae* to allow oviposition. Insects that did not oviposit within 24 h were discarded from data analysis.

### Treatments

A first set of comparisons was conducted to test whether odours from aphids or plants are attractive to ladybird females. All odour sources were confined in glass chambers connected to the olfactometer device (see “Behavioural observations”). The following treatments were performed:

- *Aphis fabae* infested plant (IP), obtained by exposing all plant stems and leaves to 200 juvenile and adult aphids for 48 h.
- *Aphis fabae* (APH) in a cage containing 200 juvenile and adult aphids. The cage consisted of a cylinder made of mesh (Ø = 28 mm; volume = 50 ml) positioned in the middle of a glass chamber.
- *Aphis fabae* honeydew (HON) obtained by positioning four plants as in treatment a) above a Parafilm foil (length = 300 mm; width = 100 mm) for 48 h. The Parafilm with the honeydew was positioned in the middle of a glass chamber.
- Clean plant (CP).
- Control (AIR), obtained by adding 200 ml tap water inside the glass chamber to render the humidity of the control chamber similar to that of the treatments.
  In the second set of comparisons, the hypothesis that the plant itself might release synomones induced after the attack by aphids was tested. The treatments consisted of:
- *Aphis fabae* infested plant (IP), as in a).
- *Aphis fabae* and clean plant (CP + APH), obtained by positioning a cage with aphids as in b) close to a clean plant. The cage was attached to a wooden holder inserted into the soil. Care was taken to prevent the aphid honeydew from contacting the plant tissues.
- Injured plants (IP -APH), obtained as in a), but aphids were carefully removed 30 min before the bioassay, both by washing the plant with tap water and using a fine paintbrush.
- Clean plant (CP), non-infested by aphids. If CP was associated with IP-APH, the plant was washed with tap water before the bioassay.
  In the third set of comparisons, we tested the duration of attractiveness of the plant attacked after aphid removal. The treatments consisted of:
- Injured plants (IP -APH), obtained as in h) and exposed respectively 24, 48, 72 or 96 h after washing.
- Clean plant (CP), non-infested by aphids, washed and exposed respectively 24, 48, 72 or 96 h after washing.
  Given that only *H. axyridis* responded positively to infested plants with the aphids carefully removed, the bioassays using j) and k) treatments were only conducted with this ladybird species.
  In the fourth set of comparisons, we tested whether the source of plant attractiveness is local or systemic. Treatments consisted of:
- Locally infested plant (IP -CLIP), obtained by exposing the lower surface of the 4^th^ leaf to 50 mixed instars aphids inside a clip cage for 48 h. Clip cage consisted of a 5-cm diameter x 1.0-cm height modified Petri dish, with the rim covered by a sponge ring and the bottom ventilated through a mesh-covered hole. The dish was supported by a hairpin attached to a wooden holder inserted into the soil. The clipped leaf was excised with forceps just before the bioassay.
- Uninfested plant (CP -CLIP), obtained by clipping an untreated 4^th^ leaf with a clip cage previously described in l). Similarly, the clipped leaf was excised with forceps just before the bioassay.

After the end of the treatment period, plants were transferred to the bioassay room.

### Behavioural observations

Female responses to volatile chemicals from different treatments were investigated using a Y-tube olfactometer made of a polycarbonate body (stem = 185 mm; arms = 125 mm at 150° angle; internal height = 10 mm; internal width = 20 mm) sandwiched between two glass plates (height = 5 mm) (Supplementary Figure S2). A pressurized tank provided a stream of medical-grade clean air (N2:O2 = 80:20) humidified by bubbling through a water jar and regulated in each arm to approximately 0.5 l min^−1^ using flowmeters. Plastic tubes (internal Ø = 8 mm) were used to connect the device parts. The device was illuminated from above with two 22-W cool white fluorescent tubes and from below by an infrared source (homogeneous emission of wavelengths at 950 nm provided by 108 LEDs). Before entering the olfactometer arms, each air stream passed through a cylindrical glass chamber (Ø = 120 mm; height = 520 mm) with an O-ring sealed middle joint, containing different odour sources (see section “Treatments”). The stimuli were randomly assigned at the beginning of the bioassays and were reversed after testing three ladybird females. At every switch, the polycarbonate olfactometer was cleaned with water and detergent, and all glass parts were exchanged for clean parts. Glass parts were cleaned with acetone and heated overnight at 180 °C. Ladybird females were singly introduced into the Y-tube olfactometer at the entrance of the stem and allowed to move freely for 5 min. A monochrome CCD video camera fitted with a 12.5–75 mm/F 1.8 zoom lens was used to record the insect behaviour. The camera lens was covered with an infrared pass filter to remove visible wavelengths. Analogue video signals from the camera were digitized by a video frame grabber^59^. Digitized data were processed by XBug, a video tracking and motion analysis system^60^. Insect response was measured in terms of residence time, i.e., the time spent by the female in each arm during the entire bioassay. The Y-tube olfactometer bioassays were carried out as paired choices, in which odour sources were always tested versus clean plants or air used as control.

The equality of the air flow inside the Y-tube olfactometer, recorded through two flowmeters as explained above, was additionally checked by observation of the flow, made visible using dry ice. In addition, to evaluate possible bias, preliminary bioassays were conducted using clean air in both olfactometer arms and by analysing the behaviour of the ladybird *H. axyridis*. No biases were recorded.

Bioassays were conducted daily from 09:00 to 17:00 under controlled conditions (26 ± 1 °C; 50 ± 5% R.H.). Twenty-nine to forty-three replicates were conducted for each treatment. Females that did not move and those that did not exhibit any choice for at least one olfactometer arm during the first 3 min of the bioassay, were discarded from the analysis. The percentage of time spent by a female in each arm of the olfactometer (Residence time) was calculated over the total experiment time and logit transformed for the analysis. The number of active ladybird females, i.e. females that walked into one or both olfactometer arms, was scored, and the percentage was calculated over the total number of replicates. Generalized Linear Models (GLMs) were fitted to test the effects of treatment vs. control arm within each source of volatiles per each ladybird species on the residence time (GLMs with Gaussian error distribution) and on the active females (GLMs with binomial error distribution). The “insect identity” effect was included as random effect in generalized mixed effect models and its relevance was tested by means of likelihood ratio test^61^. The presence of the random effect was never justified, therefore we run GLMs with only fixed effects. Overall there were four blocks of models, one for each set of comparisons (see “Treatments”). Significance of the treatments higher than controls was evaluated through planned orthogonal contrasts^62, 63^.

### Ovarian dynamics observations

To test whether volatiles from prey and plants affect the trade-off between reproduction and body mass, females treated as above (see “Insects”) were exposed to odour sources from either an infested or a clean *V. faba* plant. For each experimental setup, a total of 12 females, of both ladybird species, were individually positioned in a clean Petri dish (Ø = 90 mm) with a bottom containing filter paper (Filter-Lab Filtros Anoia 1300/80) moistened with 2 ml tap water (Supplementary Figure S3). Each dish was provided with a hole in the lid connected by means of a PE tube (Ø = 3 mm) to the side of a unique Petri dish (Ø = 90 mm), which had been modified with 12 holes in its side, to connect all of the Petri dishes. An additional hole was provided at the bottom and connected by a PE tube (Ø = 6 mm) to a glass chamber (Ø = 120 mm; height = 520 mm). A stream of clean air, humidified by bubbling through a water jar, was provided by a pump and regulated at approximately 2 l min^−1^. The device was illuminated from above by six 32-W white fluorescent tubes. The experiment began daily at 8:00 and groups of females were collected after 0, 6, 12, 24 and 48 h from the experiment set-up. Females were dissected, and their ovaries were gently extracted and treated for 30 m with Evan’s Blue 0.025%, which allows selective staining of resorptive oocytes. These specimens were observed under a stereomicroscope (as in ref. 39). The oocytes were classified as fully developed, undeveloped or resorptive. The different treatments were rotated within experimental setups and were replicated 6 to 8 times.

An additional experiment was performed to investigate the speed of new oogenesis in *H. axyridis* and *O. conglobata* after a period of starvation. Females of both ladybird species ready to oviposit were isolated in Petri dishes and starved for 72 hours to allow them to resorb all their eggs. Next, they were fed an excess of *A. fabae* (approximately 300 aphids were maintained constant during the feeding trial) and dissected 24 and 48 hours later. The ovarian dynamic was investigated by observing the first oocyte per each ovariole, for a total of 16 ovarioles (i.e. 8 per ovary) randomly selected per insect. The different treatments were replicated 8 to 10 times.

For both experiments, the number of immature oocytes (undeveloped or resorptive) or mature oocytes divided by the total number of ovarioles was arcsine square root transformed before the analysis. For the first experiment, linear regression was performed to analyse the relationship between immature oocytes and the following variables: coccinellid species, plant treatment, hours from set-up (aphids removed) and the interactions between these variables. To identify the minimum adequate model, we used backward selection, starting with a model that included the coccinellid species, plant treatment, hours, and all interactions between the variables. For the second experiment, linear regression was performed to analyse the relationship between mature oocytes and the following variables: coccinellid species, hours from set-up (aphids added), and the interactions between the variables. The minimum adequate model was identified using backward selection. For both experiments, model selection was performed based on the Akaike Information Criterion (AIC)^64^. All data were analysed using the R statistical environment^65^.

## Electronic supplementary material

Supplementary Info
