# Supplementary material for: Behavioural and physiological responses to prey-related cues reflect higher competitiveness of invasive vs. native ladybirds
Source: Sci Rep. 2017 Jun 16;7:3716. doi: 10.1038/s41598-017-03471-9 (PMC5473873; doi:10.1038/s41598-017-03471-9)

BEHAVIOURAL AND PHYSIOLOGICAL RESPONSES TO PREY-RELATED CUES REFLECT HIGHER COMPETITIVENESS OF INVASIVE VS. NATIVE LADYBIRDS

Gabriele Rondoni, Fulvio Ielo, Carlo Ricci, Eric Conti

Supplementary Figure S1: Percentage (mean ± SE) of immature oocytes in *Harmonia axyridis* and *Oenopia conglobata*, when exposed to odour sources from an infested or a clean plant, at 0, 6, 12, 24 and 48 hours from the diet removal.

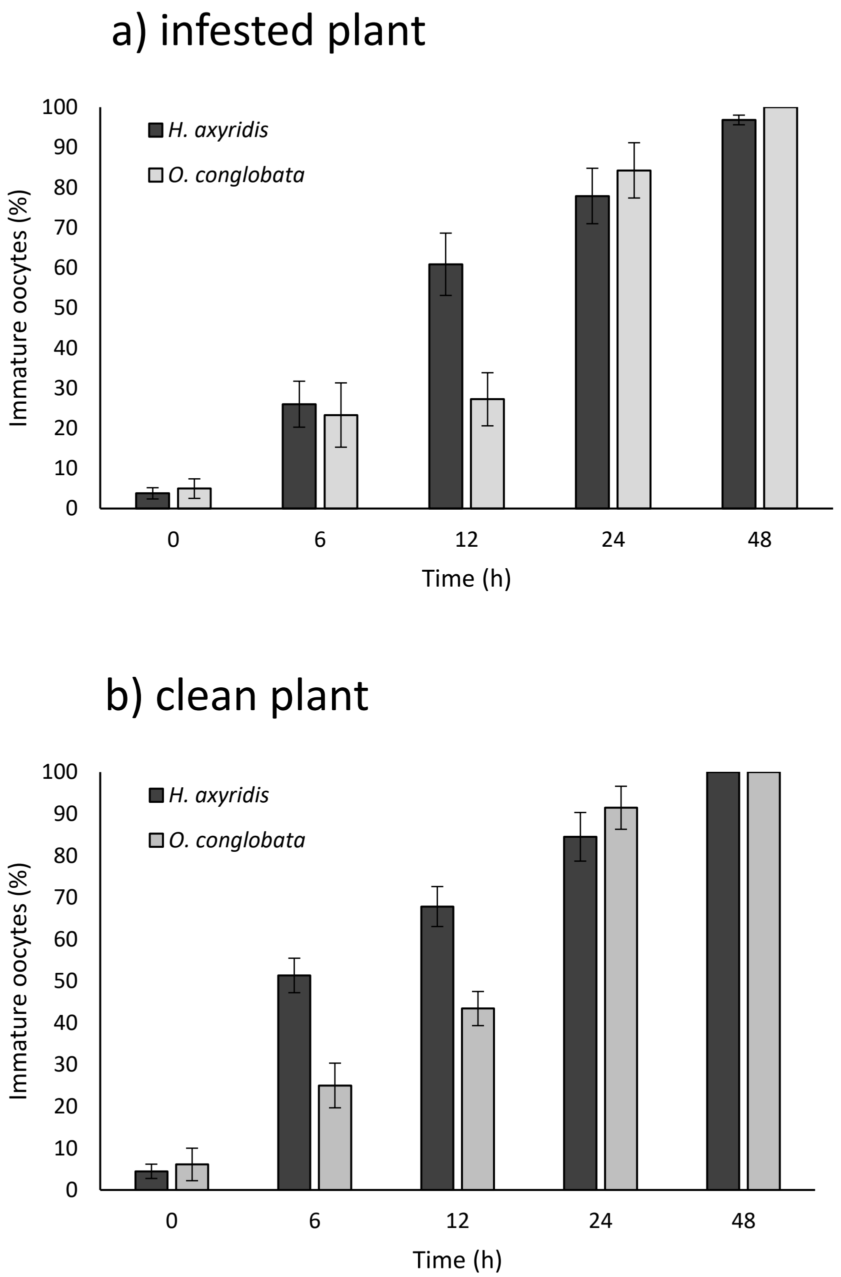

Supplementary Table S1: Summary of the hypotheses tested within the generalized linear model fitted to the data related to the first set of behavioural comparisons. GLM describes the effect on the residence time of treatment vs. control arm within each source of volatiles per each ladybird species.

Planned comparisons	Estimate	SE	z value	P	
HA: IP vs AIR	2.12	0.47	4.47	<0.001	***
HA: APH vs AIR	1.05	0.47	2.22	0.013	*
HA: HON vs AIR	0.99	0.43	2.29	0.011	*
HA: CP vs AIR	-0.81	0.47	-1.71	0.956	ns
OC: IP vs AIR	0.48	0.44	1.10	0.137	ns
OC: APH vs AIR	-0.15	0.48	-0.31	0.622	ns
OC: HON vs AIR	0.47	0.43	1.08	0.139	ns
OC: CP vs AIR	0.25	0.47	0.54	0.296	ns

Signif. codes: '***' P < 0.001, '*' P < 0.05, 'ns' P < 1

Supplementary Table S2: Summary of the hypotheses tested within the generalized linear model fitted to the data related to the first set of behavioural comparisons. GLM describes the effect on the active females of treatment vs. control arm within each source of volatiles per each ladybird species.

Planned comparisons	Estimate	SE	z value	P	
HA: IP vs AIR	2.09	0.82	2.54	0.006	**
HA: APH vs AIR	0.98	0.59	1.67	0.048	*
HA: HON vs AIR	0.57	0.63	0.91	0.181	ns
HA: CP vs AIR	-0.92	0.62	-1.47	0.929	ns
OC: IP vs AIR	0.00	0.75	0.00	0.500	ns
OC: APH vs AIR	1.64	0.84	1.94	0.026	*
OC: HON vs AIR	0.87	0.61	1.43	0.077	.
OC: CP vs AIR	-0.37	0.62	-0.61	0.729	ns

Signif. codes: '**' P < 0.01, '*' P < 0.05, '·' P < 0.1, 'ns' P < 1

Supplementary Table S3: Summary of the hypotheses tested within the generalized linear model fitted to the data related to the second set of behavioural comparisons. GLM describes the effect on the residence time of treatment vs. control arm within each source of volatiles per each ladybird species.

Planned comparisons	Estimate	SE	z value	P	
HA: IP vs CP	1.70	0.46	3.71	<0.001	***
HA: IP-APH vs CP	1.20	0.47	2.57	0.005	**
HA: CP+APH vs CP	0.91	0.46	1.98	0.024	*
OC: IP vs CP	1.22	0.42	2.92	0.002	**
OC: IP-APH vs CP	0.22	0.44	0.49	0.311	ns
OC: CP+APH vs CP	0.40	0.46	0.87	0.191	ns

Signif. codes: ‘***’ P < 0.001, ‘**’ P < 0.01, ‘*’ P < 0.05, ‘ns’ P < 1

Supplementary Table S4: Summary of the hypotheses tested within the generalized linear model fitted to the data related to the second set of behavioural comparisons. GLM describes the effect on the active females of treatment vs. control arm within each source of volatiles per each ladybird species.

Planned comparisons	Estimate	SE	z value	P	
HA: IP vs CP	2.09	0.82	2.54	0.006	**
HA: IP-APH vs CP	1.80	0.84	2.16	0.015	*
HA: CP+APH vs CP	0.84	0.59	1.42	0.078	.
OC: IP vs CP	0.57	0.63	0.91	0.181	ns
OC: IP-APH vs CP	0.00	0.69	0.00	0.500	ns
OC: CP+APH vs CP	0.42	0.65	0.64	0.260	ns

Signif. codes: ‘**’ P < 0.01, ‘*’ P < 0.05, ‘·’ P < 0.1, ‘ns’ P < 1

Supplementary Table S5: Summary of the hypotheses tested within the generalized linear model fitted to the data related to the third set of behavioural comparisons. GLM describes the effect on the residence time of treatment vs. control arm within each source of volatiles per each ladybird species.

Planned comparisons	Estimate	SE	z value	P	
HA: IP-APH24h vs CP	0.74	0.39	1.88	0.030	*
HA: IP-APH48h vs CP	-0.34	0.40	-0.84	0.799	ns
HA: IP-APH72h vs CP	-0.07	0.40	-0.17	0.569	ns
HA: IP-APH96h vs CP	0.35	0.38	0.92	0.178	ns

Signif. codes: '*' P < 0.05, 'ns' P < 1

Supplementary Table S6: Summary of the hypotheses tested within the generalized linear model fitted to the data related to the third set of behavioural comparisons. GLM describes the effect on the active females of treatment vs. control arm within each source of volatiles per each ladybird species.

Planned comparisons	Estimate	SE	z value	P	
HA: IP-APH24h vs CP	2.16	1.10	1.96	0.025	*
HA: IP-APH48h vs CP	-0.68	0.69	-0.99	0.838	ns
HA: IP-APH72h vs CP	-0.44	0.95	-0.46	0.678	ns
HA: IP-APH96h vs CP	0.19	0.62	0.31	0.379	ns

Signif. codes: '*' P < 0.05, 'ns' P < 1

Supplementary Table S7: Summary of the hypotheses tested within the generalized linear model fitted to the data related to the fourth set of behavioural comparisons. GLM describes the effect on the residence time of treatment vs. control arm within each source of volatiles per each ladybird species.

Planned comparisons	Estimate	SE	z value	P	
HA: IP-CLIP vs CP-CLIP	0.81	0.33	2.42	0.008	**
OC: IP-CLIP vs CP-CLIP	-0.03	0.32	-0.10	0.538	ns

Signif. codes: ‘**’ P < 0.01, ‘ns’ P < 1

Supplementary Table S8: Summary of the hypotheses tested within the generalized linear model fitted to the data related to the fourth set of behavioural comparisons. GLM describes the effect on the active females of treatment vs. control arm within each source of volatiles per each ladybird species.

Planned comparisons	Estimate	SE	z value	P	
HA: IP-CLIP vs CP-CLIP	1.21	0.85	1.43	0.077	.
OC: IP-CLIP vs CP-CLIP	-0.70	0.61	-1.15	0.876	ns

Signif. codes: ‘·’ P < 0.1, ‘ns’ P < 1

Supplementary Table S9. Regression coefficients, standard errors and significance for variables retained in the best-fit model describing the relationship between the speed of new oogenesis and (i) time from experiment set-up (0, 24, 48 h); (ii) coccinellid species (*Harmonia axyridis* or *Oenopia conglobata*); and (iii) interaction between time and coccinellid species.

Variable	(Level)	Coeff	SE (coeff)	P
Intercept		0.06	0.08	0.422
Time	(24 h)	0.46	0.10	< 0.001
	(48 h)	0.72	0.10	< 0.001
Species	(*O. conglobata*)	0.06	0.10	0.560
Time:species	(24 h):(*O. conglobata*)	-0.30	0.14	0.038
	(48 h):(*O. conglobata*)	-0.09	0.14	0.515

Supplementary Figure S2. Schematic of the Y-tube olfactometer device. A= pressurized air tank; P = pressure gauge; F = flowmeter; H = humidifier; C = glass chamber containing the odour sources; O = Y-tube olfactometer.

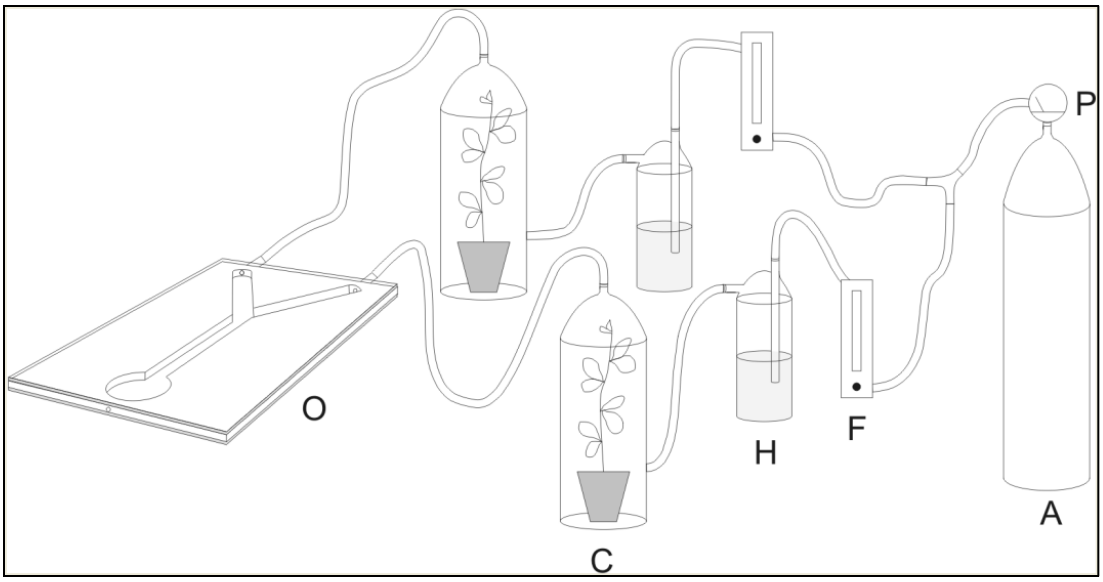

Supplementary Figure S3. Schematic of the device used to undertake the ovarian dynamic observations. P = pump; F = flowmeter; H = humidifier; C = glass chamber containing the odour sources; S = splitter; D = petri dishes, each containing one ladybird female.

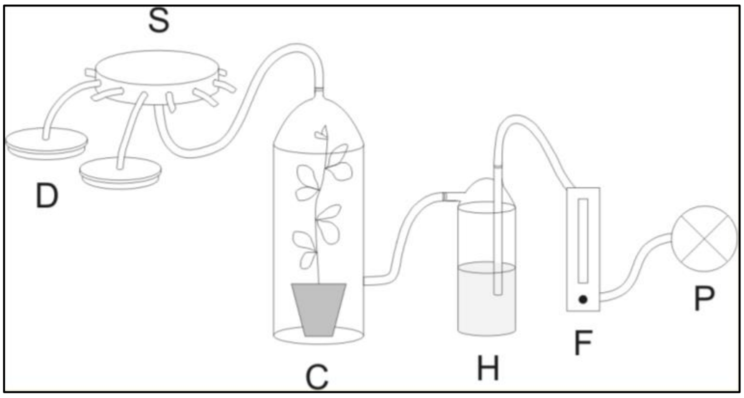

Supplement: Supplementary file 1 — Supplementary Info [file 41598_2017_3471_MOESM1_ESM.pdf]
